# Canada Cancer Clinicians’ Perceptions of Palliative Care in Pancreatic Cancer: A National Survey

**DOI:** 10.3390/curroncol33020113

**Published:** 2026-02-13

**Authors:** Kamesha Valoo, Brigitte N. Durieux, Kajamathy Subramaniam, Ting Du, Samara Perez, James Downar, Karin Fink, Justin J. Sanders

**Affiliations:** 1 Department of Family Medicine, McGill University, Montreal, QC H3S 1Z1, Canada; 2 Research Institute of the McGill University Health Centre, Montreal, QC H4A 3S9, Canada; 3 Department of Oncology, McGill University, Montreal, QC H4A 3T2, Canada; 4 Division of Palliative Care, University of Ottawa, Ottawa, ON K1N 5C8, Canada

**Keywords:** palliative care, pancreatic neoplasms, referral and consultation, surveys and questionnaires, delivery of healthcare

## Abstract

(1) Background: Pancreatic cancer causes significant morbidity and mortality, and guidelines recommend early integration of palliative care alongside active treatment. As access to palliative care is often mediated by oncology clinicians, this survey sought to understand these clinicians’ perspectives on barriers and facilitators to clinician palliative care referral and patient palliative care access. (2) Methods: An online survey was distributed via community and academic institutions across Canada to oncology clinicians who were able to refer a pancreatic cancer patient to palliative care (e.g., oncology nurses, medical oncologists). (3) Results: Of 134 clinicians from ten provinces, almost all (97%) believe that palliative care is ‘very important’ in pancreatic cancer care. The greatest perceived barriers to patient palliative care access included patient-family reluctance to accept palliative care (73%), lack of knowledge about services (50%), and lack of support from other healthcare professionals (40%). Perceived barriers to referring included patient-family reluctance to accept palliative care (57%) and lack of available specialist services (58%). Most providers suggested patient education as a strategy to overcome barriers (85%). (4) Conclusions: Perceptions about public acceptance may preclude oncology clinicians from referring to palliative care, despite nearly universal belief in its importance. The findings highlight service and education gaps for future intervention.

## Simple Summary

Pancreatic cancer is an aggressive disease and a leading cause of cancer death; as such, people with pancreatic cancer benefit from palliative care early and alongside treatment. However, most are referred to palliative care by their oncology clinician, whose perceptions of palliative care can impact the timing and likelihood of referral. While the literature has previously explored patient–provider perceptions of palliative care, we do not know what barriers to referral and access are the most notable for pancreatic cancer providers in Canada. In this study, we surveyed oncology clinicians across Canada to understand perceived barriers and facilitators to referral and access; participants reported perceived patient-family reluctance to accept palliative care, lack of knowledge about palliative care services and lack of access to palliative care services as the main barriers and patient education the main facilitator. These insights highlight palliative care service expansion and education as areas for improving guideline-concordant pancreatic cancer care.

## 1. Introduction

While pancreatic cancer is the 11th most common diagnosed cancer in Canada, it is the third most common cause of cancer-related death in Canada [1]. There has been minimal progress in early detection and new treatments for pancreatic cancer [2,3], and pancreatic cancer deaths have been relatively stable in Canada since the year 2000 [1,4]. At present, there is no available screening test for early detection [5], and most cases are diagnosed at a late stage. Estimations for 2024 postulated that approximately 7100 Canadians would be diagnosed with pancreatic cancer, and of those, 6100 would die [1]. Pancreatic cancer is associated with significant physical and psychological morbidity [6,7]. Fatigue, weight loss, abdominal pain, and depression are common symptoms [8]. Other medical complications, such as obstructive jaundice or cholangitis, can lead to increased hospital admissions and the need for broad multi-disciplinary team management [7]. These complications place a unique burden on patients, caregivers, and the healthcare system.

Evidence supports the benefit of palliative care for people affected by pancreatic cancer [7,9,10]. Palliative care is defined as the provision of active holistic care for persons experiencing serious health-related suffering due to severe illness and especially for those near end of life [11]. In Canada, it is a recognized medical specialty in all but one province and palliative care clinicians focus on improving quality of life through expert symptom management and serious illness communication. A 2022 nurse-led palliative care intervention in pancreatic cancer showed a trend towards improvement on patient self-report tools for physical, emotional, social, and functional subscales [7]. Temel and colleagues found that early integrated palliative care alongside routine oncologic care led to improved quality of life in patients with lung and non-colorectal gastrointestinal (GI) cancers [12]. A 2023 study of early palliative care for pancreatic cancer patients demonstrated both improved quality of life and reduced symptom burden for patients with metastatic disease [9]. Additionally, a pancreatic cancer study in 2021 found early palliative care referrals were associated with decreased emergency department utilization [10]. As such, the American Society of Clinical Oncology recommends early integration of palliative care for patients with both locally advanced and metastatic pancreatic cancer, preferably at the first oncology visit [13,14].

The burden of referral to palliative care falls principally on oncology clinicians, who must make decisions about referral appropriateness and timing. Though palliative care is recommended for pancreatic cancer patients from an early stage in disease trajectory, we know little about the perspectives on palliative care amongst those who refer them. Some studies have highlighted oncologist-related barriers to palliative care access for cancer patients generally [15,16], but lack specific data for pancreatic cancer patients, a population with disproportionate need. Therefore, this study aims to explore perceptions of palliative care amongst oncology clinicians for their patients with pancreatic cancer. By exploring these perceptions, we hope to identify opportunities to improve palliative care access for this population.

## 2. Materials and Methods

### 2.1. Study Design

This was a cross-sectional survey of pancreatic cancer oncology clinicians across Canada, reported in accordance with the CROSS checklist for reporting survey studies [17] in Appendix A.1. Ethics approval for this study was obtained from the Research Ethics Board of the McGill University Health Centre (protocol #2024-9580, initial approval on 31 October 2023, most recent amendment approval on 28 August 2024).

### 2.2. Survey Development and Software

The survey was adapted from a prior survey distributed to gynecologic oncologists, aimed at evaluating their perceptions of palliative care for their patients [18]. This survey included 37 items surrounding provider demographics, practice-related information, and training and experience, as well as questions about interventions perceived as helpful in addressing barriers (6 items, assessed via responses of helpful, somewhat helpful, or not helpful), provider perceptions of palliative care (8 items, assessed via Likert scale ranging from (1) strongly agree to (5) strongly disagree), perceived barriers (5 items, Likert scale), and proposed end-of-life quality measures (5 items, Likert scale) [18].

Adaptations made to the survey included the addition of demographic questions specific to the Canadian health context (Province, Urban/Rural), the re-wording of questions to pertain to the pancreatic cancer setting, and the duplication of question sets around attitudes and barriers/facilitators to separate difficulties related to patient access from difficulties related to physician referral. The adapted survey was pre-tested by an oncology nurse as well as a medical oncologist to ensure clarity, accuracy, and relevance; no major content edits were identified. The final survey is described in *Data Collection* and is available in Appendix A.2.

We used REDCap (Research Electronic Data Capture, Nashville, TN, USA), a secure online application for creating and distributing surveys [19]; a link to the survey was distributed to potential participants via email. The survey was voluntary, available in French or English, and anonymous. Compensation was provided to respondents in the form of a $10 e-gift certificate.

### 2.3. Survey Participants and Distribution

The survey targeted various oncology clinicians including oncology nurses, medical oncologists, surgical oncologists, radiation oncologists, and GI specialist oncologists. To be included in the study, participants had to be either an oncology physician or nurse with experience in the care of pancreatic cancer patients in Canada and be fluent in either English or French. The survey was initially distributed via various representative national organizations across Canada. Additional attempts at outreach were made by contacting university-affiliated oncology department heads across Canadian universities. Eventually, outreach was extended to include general practitioner (GP) oncologists, a previously overlooked group.

### 2.4. Data Collection

The questionnaire comprised 56 questions and was divided into four main sections: demographic information and clinical experience, attitudes towards palliative care, barriers to access and referral of palliative care, and open-ended comments. We collected demographic information such as gender, age, race, and province of practice. We assessed clinical experience with questions about profession, number of years of practice, and average number of pancreatic cancer patients seen per year. The survey was open between 7 February 2024 and 8 November 2024, and is available in full in Appendix A.2.

### 2.5. Data Cleaning

Preliminary data was downloaded from REDCap as a .csv file and analyzed via Microsoft Excel; we cleaned the data through multiple steps, removing any incomplete surveys (n = 2), duplicate surveys from the same individual, identified by a duplicate email entered for receipt of the compensation e-gift certificate (n = 3) (we retained the survey that they took more time to complete as this was likely of higher quality), entries from careless responders (n = 2), and any entries associated with nonsensical or non-human text (n = 0).

We looked for careless responders by checking variance [20], flagging any respondent who had no variance in at least 3 of our 6 multi-item questionnaires (variance cases manually reviewed; n = 0 excluded). We also reviewed survey response time; after calculating quartiles of response times and finding several notable but reasonable outliers and lack of a normal distribution, we did not use statistical methods to exclude participants. Rather, given the number of items involved in the survey (which, from practice tests, was expected to reasonably take about 15 min), we presumed that any respondents who completed the survey in under four minutes were likely careless responders (n = 2 excluded). Our final dataset comprises 134 quality-checked surveys from distinct participants.

### 2.6. Data Analysis

We report participant response frequencies via respondent counts and percentages. In some cases, we collapsed variables based on what made sense for comparison (i.e., ‘agree’ or ‘strongly agree’ were considered as ‘agree’). In an initial exploratory analysis, we used Chi-squared tests to determine if there were significant statistical associations between demographic- and profession-related variables and survey responses, but we were not sufficiently powered to report associations in the dataset due to an insufficient number of samples across variables or small effect sizes. We report response frequencies, as these remain informative, as well as themes from free-text responses to open-ended survey questions with illustrative examples of provided comments. As free-text survey responses did not constitute sufficiently rich data for formal qualitative analysis, responses were analyzed via the authors (K.V., B.D., J.S.) collating and reviewing responses to each free-text question; responses (often a single phrase or sentence) were read, sorted into common groups, and the groupings reported as free-text themes.

## 3. Results

### 3.1. Sample Characteristics

Our dataset included completed surveys from 134 respondents. The majority of respondents were of female gender (81%), and the most common race reported was white (66%). Forty-seven percent of respondents were from Central Canada (37% from Ontario); and eighty-one percent of respondents practiced in urban areas, as opposed to 19% practicing in rural settings. Sixty-three percent of respondents were oncology nurses. In terms of experience, 30% of respondents had 0–5 years of practice, and 28% had 6–10 years. See Table 1 for participant demographics.

### 3.2. Survey Responses

#### 3.2.1. Attitudes Toward Palliative Care

Almost all (98%) respondents considered palliative care to be “very important” in the care of pancreatic cancer patients. Additionally, 66% of respondents were at least somewhat satisfied with the quality of palliative care provided to their patients (20% were very satisfied). Only 8% of respondents agreed that “palliative care begins where medical oncology ends”, and 90% of respondents agreed that all advanced pancreatic cancer patients should receive concurrent palliative care even if they are receiving anti-tumor therapies. See Table 2 for results.

#### 3.2.2. Barriers and Facilitators to Access and Referral

When considering barriers to accessing palliative care services for patients, the majority of respondents identified patient and family reluctance to accept palliative care as a barrier (72%). This was followed by a lack of knowledge about palliative care services (51%), and a lack of support from other healthcare professionals (40%). Patient and family reluctance was also identified as a main barrier to sending referrals to palliative care services (55%), along with lack of available specialist palliative care services (57%). When asked about strategies used to try and overcome these barriers, most providers indicated that they educate patients about palliative care services (84%). Many providers indicated that they rely on increased collaboration with other healthcare professionals to try to overcome these barriers (68%). See Table 3 for results.

#### 3.2.3. Free-Text Responses

We collected open-ended comments about other perceived barriers for providing palliative care for pancreatic cancer patients. Five main themes were prominent amongst the open-ended comments submitted: lack of resources within the healthcare system, lack of collaboration amongst healthcare providers, patient perceptions of palliative care, restrictive exclusion criteria for accessing palliative care, and unclear referral guidelines. See Table 4 for results.

Free-text participant responses indicated complexity regarding patient-family reluctance to accept palliative care; some respondents noted patient perceptions of palliative care (i.e., a signal of immediate death, need to stop anti-cancer therapy), and others mentioned exclusion criteria to palliative care programs supporting patients’ perceptions (i.e., lack of resources, some settings have no concurrent palliative care option and indeed require patients to stop anti-cancer therapy).

## 4. Discussion

We conducted a survey of oncology clinicians who care for those people with pancreatic cancer to learn their perspectives of palliative care for this population. Nearly all participants considered palliative care to be very important in the care of pancreatic cancer patients, and most believed that palliative care should occur concurrently for pancreatic cancer patients still receiving anti-cancer therapies. Patient and family reluctance to the involvement of palliative care was identified as the main perceived barrier to palliative care access and referral; other important barriers included lack of knowledge about services, lack of support from other healthcare professionals, and a lack of available palliative care specialist services. Most providers suggested patient education as a strategy to overcome barriers. Free-text responses added nuance and context to survey findings around perceived barriers and facilitators.

Our survey shows that clinicians’ attitudes about palliative care in this study were aligned with current American Society of Clinical Oncology guidelines for patients with metastatic as well as locally advanced pancreatic cancer, which strongly recommend early involvement of palliative care services in patient care [13,14]. We found that patient and family reluctance to receive palliative care services was a major barrier to early palliative care access, despite its recommendation. Our findings align with results from a 2019 survey of gynecologic oncologists’ perspectives on palliative care for their patients, which identified patient factors (unrealistic expectations) as a main barrier to connecting them with palliative care services [18]. Furthermore, similar work evaluating patients with blood cancers found that patient perceptions were a major barrier to them receiving quality end of life care [19], and in the pediatric setting, parent attitudes were identified as a perceived barrier to having advanced care discussions [21]. This highlights the need for increased patient and family awareness on the benefits of palliative care and education about the role of palliative care in the context of life-limiting illnesses.

Our findings point to a potentially complex relationship between palliative care education and the reality of services offered in different settings. Despite integrated palliative care being evidence-based best care, this is not yet standard practice in many healthcare settings. For example, a study of patients with primary brain tumors revealed they were uncertain whether the implementation of palliative care would lead to cessation of other treatments [22]. An interview study of clinicians in Quebec highlighted 3-month prognosis requirements and limited beds as barriers to palliative care access [23]. A survey of clinicians in Alberta described a number of opportunity and capability barriers leading to late referrals to palliative care, which in turn contributed to patients’ perceptions of palliative care as solely end-of-life care [24]. Additionally, a survey of oncologists in Canada revealed that lack of availability of palliative care services for patients still undergoing chemotherapy, as well as biases associated with the term “palliative care”, were the main barriers to early palliative care referral [25]. This complexity makes the prospect of demystifying palliative care for patients and families difficult. Realistic inclusion and exclusion criteria should be implemented, and service and policy changes are needed to make integrated palliative care options a reality in a greater number of settings.

Health systems might consider using palliative care educational modules for patients with pancreatic cancer and their oncology clinicians in order to provide more information about palliative care and promote the need for early, integrated palliative care. Exploring patient and family acceptance of referral to palliative care in pancreatic cancer will be important as it may be that patients are willing to accept palliative care referrals, but that clinicians have misperceptions about their willingness to do so unnecessarily. Additionally, as most of our respondents were nurses, gathering nurse perspectives on palliative care for pancreatic cancer patients specifically could be an area of further research. Strong participation of nurses in our study could indicate that there is further opportunity to recruit and engage these clinicians who play an integral role in the care of pancreatic cancer patients, including care coordination and assessing symptoms and needs [6].

This survey study has limitations. First, we do not know how many oncology clinicians are practicing in Canada; hence, commenting on the level of engagement or the representativeness of the sample is a challenge. To mitigate this, we pursued multiple rounds of circulation and expanded the study population to include previously overlooked groups (GP oncologists). Second, cross-sectional surveys cannot account for changes in perspective over time and do not permit assertions regarding causality. Third, the survey may be susceptible to selection bias—we included direct outreach to universities in our recruitment strategy, which may have led to participation by those with more access to palliative care—as well as non-response bias, as those who chose to respond may have more awareness about or investment in palliative care. Lastly, although the relative distribution of Rural vs. Urban respondents reflects Canada’s population distribution [26], the lower proportion of rural and community providers meant we were not powered to assess differences in responses; the aggregated responses reported in this study thus reflect the Urban clinician reality more so than that of Rural clinicians.

## 5. Conclusions

Palliative care improves experience and outcomes for people with cancer and promotes more effective resource utilization in health systems. Therefore, ensuring access is critical in an aging population with rising cancer incidence. This survey sought to identify and understand Canadian oncology clinician perceptions of palliative care for pancreatic cancer patients. Oncology clinicians are in support of integrating palliative care alongside anti-cancer therapies but perceive patient and family perspectives of palliative care as a major perceived barrier to referrals and access. Ongoing patient and caregiver education about the known benefits of palliative care for this vulnerable patient population and skill building among clinicians to discuss palliative care are imperative next steps to facilitate early referrals to palliative care.

## Figures and Tables

**Table 1:** Survey respondent demographics (n = 134).

Characteristic	Response	Individuals (n, %)
Profession	RN (*oncology nurse*)	84, 63%
	General physician (*GP oncologist, general medical oncologist*)	12
	Specialist physician (*GI specialist, radiation oncologist*)	38
Years of Practice	0–5 years	40, 30%
	6–10 years	37
	11–15 years	24
	16–20 years	13
	>20 years	20
Patients with Pancreatic Cancer Seen Per Year on Average	0	2
	1 to 10	41
	11 to 25	50, 37%
	26 to 50	29
	>50	12
Gender	Female	108, 81%
	Male	22
	Prefer not to answer	2
	No data	2
Race	White	89, 66%
	Asian (*South Asian, East Asian, Southeast Asian*)	20
	Black	3
	Indigenous (*First Nations, Inuk/Inuit, Métis*)	2
	Middle Eastern	1
	Other (*Multiracial, Caribbean, Unspecified*)	4
	Prefer not to answer	6
	No data	9
Region/Province	Central (*Ontario, Quebec*)	63, 47%
	Western (*Alberta, British Columbia, Manitoba, Saskatchewan*)	50
	Atlantic (*Newfoundland and Labrador, Nova Scotia, New Brunswick, Prince Edward Island*)	19
Urban/Rural	Urban	108, 81%
	Rural	25
	No data	1

Percentages listed for most common response. Abbreviations: GP, general practitioner; GI, gastrointestinal.

**Table 2:** Attitudes towards palliative care (n = 134).

How important do you think palliative care is in the care of patients with pancreatic cancer?		
	Very important	131, 97.8%
	Important	2, 1.5%
	Not important	1, 0.7%
How satisfied are you with the quality of palliative care provided to patients with pancreatic cancer in your care setting?		
	Very dissatisfied	4, 3.0%
	Somewhat dissatisfied	19, 14.2%
	Neutral	22, 16.4%
	Somewhat satisfied	62, 46.3%
	Very satisfied	27, 20.1%
Palliative care begins where medical oncology ends. ^1^		
	Disagree	121, 90.3%
	Don’t know	2, 1.5%
	Agree	11, 8.2%
All advanced pancreatic cancer patients should receive concurrent palliative care even if they are receiving anti-tumor therapies. ^1^		
	Disagree	3, 2.2%
	Don’t know	10, 7.5%
	Agree	121, 90.3%

^1^ Disagree count includes responses of Disagree and Disagree Strongly; Agree count includes Agree and Agree Strongly.

**Table 3:** Barriers to patient access and clinician referral to palliative care.

What do you perceive as the main barriers to *accessing* primary palliative care for patients with pancreatic cancer? (Select all that apply)			
			n,% checked
		Patient/family reluctance to accept palliative care	97, 72.4%
		Lack of knowledge about palliative care services	68, 50.8%
		Lack of support from other healthcare professionals	54, 40.3%
		Time constraints	52, 38.8%
		Lack of referral guidelines	41, 30.6%
		Other	31, 23.1%
What do you perceive as the main barriers to *referring* patients with pancreatic cancer to specialty palliative care? (Select all that apply)			
			n,% checked
	Lack of knowledge about palliative care services		77, 57.5%
	Patient/family reluctance to accept palliative care		74, 55.2%
	Lack of referral guidelines		37, 27.6%
	Lack of support from other healthcare professionals		36, 26.9%
	Time constraints		34, 25.4%
	Other		16, 11.9%
What strategies have you used to overcome barriers to accessing and referring to palliative care? (Select all that apply)			
			n,% checked
	Increased patient/family education about palliative care		113, 84.3%
	Increased collaboration with other healthcare professionals		91, 67.9%
	Attended palliative care education/training		82, 61.2%
	Used a referral decision aid/tool		20, 14.9%
	Other		8, 6.0%

Note that respondents could check multiple boxes. Percentages represent the proportion of respondents who reported a given element.

**Table 4:** Themes observed among free-text survey responses.

Resources within the healthcare system
Examples: “…time constraints within my practice (workload measures do no account for time and care provided to these patients), wait times for the appointments” “It is frustrating to try and access outpatient palliative care at our centre, despite being a large quaternary centre with many outpatient palliative care specialists. The follow-up to these specialists is infrequent, the patients come to us with their symptoms, and we are often left to manage the symptoms of our patients…”
Collaboration amongst healthcare professionals
Examples: “We must collaborate effectively. It is counterproductive for a palliative care doctor to repeatedly report their colleagues and complain about the medical oncologist or the medical oncology team not fulfilling their duties. We must put an end to these complaints. We are all here for the patients, and together, we are stronger” “Teaching regarding palliative care and early referral to palliative care needs to occur in the residency training programs so that when physicians become staff, they have developed a comfort in referring…” “…Communication from palliative care providers is not usually very good. Many do not put notes on our EMR, even if they have access. I always send my notes, but do not feel all communicate back.”
Patient perceptions
Examples: “Misconception that palliative care means immediate death & patients are then reluctant to agree to the referral.” “The word ‘palliative’ can have strong connotations for patients with family members that passed away previously…” “They feel that if they choose palliative care that they are cut of from oncology care”
Exclusion criteria
Examples: “…specific barriers such as required LOC to be seen or followed in pall care and patients not willing to accept this requirement. Ex: LOC B requesting treatment for rapidly reversible conditions and therefore no access to pall care” “Palliative care programs that require pts stop chemo before registration on palliative care. Many patients would gladly be on PC pgms while on palliative chemotherapy for better support”
Guidelines
Example: “Lack of palliative care built into the structure and workflow (should be built in earlier for disease sites such as pancreatic cancer where time constraints do factor in)…”

Abbreviations: EMR, electronic medical record; LOC, level of care; pall care, palliative care; pts, patients; PC pgms, palliative care programs.

## Data Availability

Due to ethical restrictions and participant confidentiality, the data that support the findings of this study are not publicly available. Survey responses are anonymous and were collected using REDCap; access to de-identified data may be granted upon reasonable request to the corresponding author (Justin Sanders, justin.sanders@mcgill.ca), subject to institutional ethics committee approval (MUHC REB).

## Abbreviations

The following abbreviations are used in this manuscript:

GI	Gastrointestinal
GP	General practitioner

## Appendix A

### Appendix A.1

Survey study reporting via the CROSS checklist.

**Table A1:** Checklist for reporting survey studies (CROSS).

Section/Topic	Item	Item Description	Reported on Page #
Title and abstract			
Title and abstract	1a	State the word “survey” along with a commonly used term in title or abstract to introduce the study’s design.	p. 1
	1b	Provide an informative summary in the abstract, covering background, objectives, methods, findings/results, interpretation/discussion, and conclusions.	pp. 1–2
Introduction			
Background	2	Provide a background for the rationale of study, what has been previously done, and why this survey is needed.	p. 2
Purpose/aim	3	Identify specific purposes, aims, goals, or objectives of the study.	p. 2
Methods			
Study design	4	Specify the study design in the methods section with a commonly used term (e.g., cross-sectional or longitudinal).	p. 3
Data collection methods	5a	Describe the questionnaire (e.g., number of sections, number of questions, number and names of instruments used).	p. 3 (followed description available from initial survey paper [19])
	5b	Describe all questionnaire instruments that were used in the survey to measure particular concepts. Report target population, reported validity and reliability information, scoring/classification procedure, and reference links (if any).	Instruments not used in this manner; description of items given on p. 3
	5c	Provide information on pre-testing of the questionnaire, if performed (in the article or in an online supplement). Report the method of pre-testing, number of times questionnaire was pre-tested, number and demographics of participants used for pre-testing, and the level of similarity of demographics between pre-testing participants and sample population.	p. 3
	5d	Questionnaire, if possible, should be fully provided (in the article, or as appendices or as an online supplement).	Appendix A.2
Sample characteristics	6a	Describe the study population (i.e., background, locations, eligibility criteria for participant inclusion in survey, exclusion criteria).	p. 3
	6b	Describe the sampling techniques used (e.g., single stage or multistage sampling, simple random sampling, stratified sampling, cluster sampling, convenience sampling). Specify the locations of sample participants whenever clustered sampling was applied.	p. 3, described distribution narratively
	6c	Provide information on sample size, along with details of sample size calculation.	Denominator information unavailable to inform sample size calculation; described in limitations (p. 9)
	6d	Describe how representative the sample is of the study population (or target population if possible), particularly for population-based surveys.	Unknown; potential effect of response bias described in limitations (p. 9)
Survey administration	7a	Provide information on modes of questionnaire administration, including the type and number of contacts, the location where the survey was conducted (e.g., outpatient room or by use of online tools, such as SurveyMonkey).	High-level information given on p. 3
	7b	Provide information on the survey’s time frame, such as periods of recruitment, exposure, and follow-up days.	p. 3
	7c	Provide information on the entry process: → For non-web-based surveys, provide approaches to minimize human error in data entry. → For web-based surveys, provide approaches to prevent “multiple participation” of participants.	pp. 3–4
Study preparation	8	Describe any preparation process before conducting the survey (e.g., interviewers’ training process, advertising the survey).	N/A; p. 3 describes evolving survey distribution
Ethical considerations	9a	Provide information on ethical approval for the survey if obtained, including informed consent, institutional review board [IRB] approval, Helsinki declaration, and good clinical practice [GCP] declaration (as appropriate).	p. 3
	9b	Provide information about survey anonymity and confidentiality and describe what mechanisms were used to protect unauthorized access.	N/A; use of secure REDCap, distribution mechanism, and data cleaning processes on pp. 3–4
	10a	Describe statistical methods and analytical approach. Report the statistical software that was used for data analysis.	Analytical approach described on p. 4
	10b	Report any modification of variables used in the analysis, along with reference (if available).	p. 4
	10c	Report details about how missing data was handled. Include rate of missing items, pp. 4–5 missing data mechanism (i.e., missing completely at random [MCAR], missing at random [MAR] or missing not at random [MNAR] and methods used to deal with missing data (e.g., multiple imputation).	p. 4; missing data reported as such, not replaced.
	10d	State how non-response error was addressed.	Described in limitations (p. 9)
	10e	For longitudinal surveys, state how follow-up was addressed.	N/A
	10f	Indicate whether any methods such as weighting of items or propensity scores have been used to adjust for non-representativeness of the sample.	N/A
	10g	Describe any sensitivity analysis conducted.	N/A
Results			
	11a	Report numbers of individuals at each stage of the study. Consider using a flow diagram, if possible.	N/A; initial denominator/pool of potential participants unknown (lack of registry).
	11b	Provide reasons for non-participation at each stage, if possible.	N/A; per above.
	11c	Report response rate, present the definition of response rate or the formula used to calculate response rate.	N/A; denominator unknown.
	11d	Provide information to define how unique visitors are determined. Report number of unique visitors along with relevant proportions (e.g., view proportion, participation proportion, completion proportion).	Unique respondents determined by email address during data cleaning (p. 4); unique views/visits not collected.
	12	Provide characteristics of study participants, as well as information on potential confounders and assessed outcomes.	pp. 4–5 and Table 1
	13a	Give unadjusted estimates and, if applicable, confounder-adjusted estimates along with 95% confidence intervals and *p*-values.	N/A
	13b	For multivariable analysis, provide information on the model building process, model fit statistics, and model assumptions (as appropriate).	N/A
	13c	Provide details about any sensitivity analysis performed. If there are a considerable amount of missing data, report sensitivity analyses comparing the results of complete cases with that of the imputed dataset (if possible).	N/A; data relatively complete.
Discussion			
	14	Discuss the limitations of the study, considering sources of potential biases and imprecisions, such as non-representativeness of the sample, study design, important uncontrolled confounders.	p. 9
	15	Give a cautious overall interpretation of results, based on potential biases and imprecisions and suggest areas for future research.	pp. 8–9
	16	Discuss the eternal validity of the results.	p. 8 (discussed alignment with other research)
Other sections			
	17	State whether any funding organization has had any roles in the survey’s design, implementation, and anlaysis.	Funder acknlowledged on p. 10; funders not involved in any of these activities
	18	Declare any potential conflict of interest.	p. 10
	19	Provide names of organizations/persons that are acknowledged along with their contribution to the research.	p. 10

From: Sharma, A.; Minh Duc, N.T.; Luu Lam Thang, T.; Nam, N.H.; Ng, S.J.; Abbas, K.S.; Huy, N.T.; Marušić, A.; Paul, C.L.; Kwok, J.; Karbwang, J. A consensus-based checklist for reporting of survey studies (CROSS). *J. Gen. Intern. Med.* **2021**, *36*, 3179–3187 [17].

### Appendix A.2

Shared below is the full survey delivered to GI clinicians.

**Table A2:** Full survey.

Section	Question	Response Options
Demographic Information	Gender	(a) Male (b) Female (c) Transgender (d) Non-binary (e) Prefer not to answer
	Race	(a) Black (b) East Asian (c) Indigenous (First Nations, Inuk/Inuit, Métis) (d) Latin American (e) Middle Eastern (f) South Asian (g) Southeast Asian (h) White (i) Do not know (j) Prefer not to answer (k) Other (please specify)
	Age	(a) 18–29 (b) 30–39 (c) 40–49 (d) 50–59 (e) >60
	Province	(a) Alberta (b) British Columbia (c) Manitoba (d) New Brunswick (e) Newfoundland (f) Nova Scotia (g) Ontario (h) Prince Edward Island (i) Quebec (j) Saskatchewan
	Urban/Rural	(a) Urban (b) Rural
Clinical Experience	What is your profession?	(a) GI specialist oncologist (b) General medical oncologist (c) Radiation oncologist (d) Surgical oncologist (e) Oncology nurse (f) GP oncologist
	How many years of experience do you have in oncology?	(a) 0–5 years (b) 6–10 years (c) 11–15 years (d) 16–20 years (e) >20 years
	How many patients with pancreatic cancer do you see per year on average?	(a) 0 (b) 1–10 (c) 11–25 (d) 26–50 (e) >50
Attitudes Towards Palliative Care	How important do you think palliative care is in the care of patients with pancreatic cancer?	(a) Not important (b) Somewhat important (c) Important (d) Very important (e) Don’t know
	How confident are you in providing palliative care to patients with pancreatic cancer?	(a) Not important (b) Somewhat important (c) Important (d) Very important (e) Don’t know
	How satisfied are you with the quality of palliative care provided to patients with pancreatic cancer in your care setting?	(a) Very dissatisfied (b) Somewhat dissatisfied (c) Neutral (d) Somewhat satisfied (e) Very satisfied
	In your dealings with patients with pancreatic cancer, how often do you collaborate with… …a palliative care medical specialist? …a palliative care nurse specialist? …home-based palliative care? …an inpatient hospice? …a psychiatrist? …a psychologist?	(a) Never (b) Occasionally (c) Often
	In your dealings with patients with pancreatic cancer, how often are you directly involved with… …managing cancer pain? …managing dyspnea? …managing fatigue? …managing nausea and vomiting? …managing the complications of chemotherapy? …managing the psychological consequences of advanced cancer (e.g., depression and anxiety)? …managing delirium? …managing constipation or diarrhea? …discussing end of life preferences with patients? …directly administering end of life care to dying cancer patients? …coordinating meetings with the family of dying patients? …recommending an inpatient hospice?	(a) Never (b) Occasionally (c) Often
	Do you agree with the following statements about the management of patients with advanced cancer? …Medical oncologists should coordinate the care of pancreatic cancer patients at all stages of disease, including end of life care. …Palliative care begins where medical oncology ends. …I received good training in palliative care during my oncology fellowship (residency). …All advanced pancreatic cancer patients should receive concurrent palliative care even if they are receiving anti-tumor therapies. …The medical oncologist is the best person to coordinate the palliative care of patients with advanced pancreatic cancer. …A palliative care specialist is the best person to coordinate the palliative care of patients with advanced pancreatic cancer. …Medical oncologists should be expert in the management of the physical and psychological symptoms of advanced pancreatic cancer. …Most medical oncologists I know are expert in the management of the physical and psychological symptoms of advanced pancreatic cancer. …I am expert in the management of the physical and psychological symptoms of advanced pancreatic cancer. …Dying patients do not belong in the oncology ward. …All cancer centres should have a palliative care service. …I derive satisfaction from managing the physical symptoms of my patients. …Managing patients with advanced pancreatic cancer and dying patients depresses me. …I would rather have someone else look after my dying patients. …I am usually successful in managing my patient’s pain. …I derive satisfaction from my work managing patients with advanced pancreatic cancer and dying patients. …I feel emotionally burned out by having to deal with too many deaths. …I own a textbook of palliative care. …I read journals and papers related to the palliative care of advanced pancreatic cancer. …I am interested in participating in research in palliative treatments of advanced pancreatic cancer. …I deal with palliation in the nondying patients (“symptoms management”), but not with the palliation of the dying patient (“end of life care”). …I have a close working relationship with the palliative care (or hospice) services in my region. …Palliative care specialists “steal” patients who would otherwise benefit from medical oncology. …Palliative care (or hospice) physicians do not have enough understanding of oncology to counsel patients with advanced pancreatic cancer regarding their treatment options.	(a) Agree strongly (b) Agree (c) Do not know (d) Disagree (e) Disagree strongly
Barriers to Access and Referral of Palliative Care	What do you perceive as the main barriers to accessing primary palliative care for patients with pancreatic cancer? (Select all that apply)	(a) Lack of knowledge about palliative care services (b) Lack of referral guidelines (c) Time constraints (d) Lack of support from other healthcare professionals (e) Patient/family reluctance to accept palliative care (f) Other (please specify)
	What do you perceive as the main barriers to referring patients with pancreatic cancer to specialty palliative care? (Select all that apply)	(a) Lack of knowledge about palliative care services (b) Lack of referral guidelines (c) Time constraints (d) Lack of support from other healthcare professionals (e) Patient/family reluctance to accept palliative care (f) Other (please specify)
	What strategies have you used to overcome barriers to accessing and referring to palliative care? (Select all that apply)	(a) Attended palliative care education/training (b) Increased collaboration with other healthcare professionals (c) Increased patient/family education about palliative care (d) Used a referral decision aid/tool (e) Other (please specify)
Open-Ended Comments	What other barriers or needs do you see in providing palliative care to patients with pancreatic cancer?	
	Do you have any other comments?	
